# Book Reviews

**Published:** 1831-11

**Authors:** 


### BIBLIOGRAPHICAL NOTICES.

*Observations on Cholera, as it appeared at Port-Glasgow, during the Months of July and August* 1831. *Illustrated by numerous Cases.* By John Marshall, m.d.—8vo. pp. 86. Waugh and Innes, Edinburgh; M. Ogle, Glasgow; Curry, jun. and Co., Dublin; Whittaker and Co., London.

It rarely happens, when a practitioner has any really interesting information in his possession, that he waits for any especial provocation to induce him to take up his pen, and address the profession: but such, it appears, has been the case with Dr. Marshall: many cases of cholera having fallen under his care, the prominent symptoms of which differed from any he had before met with in this country, he felt it his duty, agreeably to his Majesty's proclamation, to send communications respecting them to the privy council. These communications were considered of so much importance, that the council sent Dr. Daun to inquire minutely into the circumstances, and report; and this gentleman was joined by Dr. Badenach.

About three weeks after Dr. Marshall had transmitted to the government an account of these cases of cholera, an anonymous attack was made upon his "character and conduct," in the Glasgow Herald, for the treatment he had adopted in them. He was also ridiculed and reflected on, as having, by his communications with the privy council and board of health, given rise to unnecessary alarm. It was asserted that the disease complained of was neither more nor less than the common cholera of the country, which annually prevails there. As far as we can judge from the pamphlet before us, and we confess that is the only source from which we have obtained any information upon the subject, Dr. Marshall did not deserve any of these aspersions: he appears to us, indeed, to have been very unjustly treated. Taking for granted the accuracy of the reports here given, we cannot but express our surprise that any man of ordinary experience and observation could for a moment look upon these cases as examples of the *common* cholera of this country. Dr.

Marshall, in our opinion, was not only "justified in calling the attention of the authorities to the symptoms of the disease at that time prevailing in Port-Glasgow," but he would have been guilty of a gross dereliction of his duty, as a man and a physician, if he had withheld so important a communication.

Dr. Marshall does not mean to assert that the cholera of India or Russia has been *epidemic* in Great Britain: all he contends for is, that cholera has, during the present year, appeared at a season and in a form hitherto most uncommon, if not unknown; and that, wherever it has so appeared, cases, not always *insulated*, have occurred, bearing in every feature, except their fatality, the strongest resemblance to those detailed by CURTIS, ANNESLEY, SMITH, SCOTT, and other writers on the diseases of India, and also to those described by Dr. SOKOLOFF at Orenburg; "and that, therefore, come when it may as an epidemic, the public have no reason to look forward to its arrival as to that of a mysterious pestilence, *utterly unknown* to British constitutions and British physicians, but as a disease which has been already seen, grappled with, and in all ordinary circumstances subdued."

We do not think it necessary to enter into a detailed account of the twenty-four cases of cholera that are related by Dr. Marshall, as, in his "concluding observations," he briefly refers to the leading characters of the disease which he thinks justify the opinions he has adopted. In all the cases but one there was a burning pain between the scrobiculis cordis and umbilicus, and this symptom Mr. Annesley (Diseases of India, p. 38,) asserts to be the constant, and even *invariable* pathognomonic symptom; "and which," he adds, "I therefore consider as *particularly characteristic* of the epidemic cholera." The appearance of the discharges from the alimentary canal was similar to that described by Mr. Scott, in his "Report of the Epidemic Cholera of India," Madras, 1824. Two of the least variable symptoms met with by Dr. Marshall were the "peculiar state of collapse to which the circulating power was reduced by the very first attack of the disease, and the equally peculiar state of the skin and countenance," described by writers on the Indian cholera. In every one of these cases also there was suppression of urine;* and in most "this symptom continued after reaction was established, when a small quantity of dark-coloured urine was voided." As in none of the cases which Dr. Marshall saw venesection was thought advisable, no opinion could be formed of the state of the blood, further than it appeared, when abstracted by leeches, which were freely used, black and ropy, as described by Indian authors.

Upon what diagnostic of the Indian disease, then, can we depend, asks Dr. M., which is not to be found in some one, if not all, of the foregoing cases?

The remedies employed were chiefly brandy, opium, and calomel; heat to the surface, frictions, &c. No narcotic effect followed the free use of opium; neither did ptyalism take place from calomel. These drugs were always used in combination, in the form of pill, and it was observed how very rarely they were ejected from the stomach, even when vomiting was most urgent; but when calomel was used in powder, it was very seldom retained. Dr. Marshall mentions the very marked advantage and relief to the patient which uniformly followed the application of a bandage, as tight as it could be borne, round the body, from the waist downwards: he generally used a strip of stout flannel, two or three yards long, and of a suitable breadth. In one case, where vomiting, purging, and agonizing pain of the bowels subsided on the application of the bandage, they immediately reappeared on its being removed by the impatience of the sufferer, and were again relieved when it was replaced. Only one of the cases related by Dr. Marshall terminated fatally; but this fact does

* Vide Drs. BARRY and RUSSELL's Report of Russian Cholera; London Med. and Phys. Journal, October 1831, p. 357.

not militate against his opinions. It has been supposed, by different and competent authorities, that, if ever the Indian cholera did visit this country, it would be much less destructive than it has been in other parts of the world.

---

*A Treatise on Auscultation, illustrated by Cases and Dissections.* By ROBERT SPITTAL, lately Physician's Assistant, and now House Surgeon, Royal Infirmary of Edinburgh; President of the Plinian Natural History Society, &c.—8vo. pp. 280; Plates. Grant and Sons, Edinburgh; Hurst and Co. London; and Curry, Dublin.

IN the year 1829, the Harveian Medical Society of Edinburgh intimated, as the subject of their prize essay, "the Diagnostic Properties of the Stethoscope, illustrated by Dissections:" the author of this work was the successful competitor, and this mark of the approbation of the distinguished members of that society has been the chief inducement for submitting this, the Harveian Essay, to the profession. It is confessed that the experienced stethoscopist will meet with little that is new in this work; but the author hopes that, having endeavoured to include in it all that has been made known on this subject, from the time of its great inventor to the present, and given a few practical illustrations, he will have rendered this portion of his essay worthy the notice of the student in auscultation; while it is trusted that the pathological department will be of some value, from the cases and drawings which have been furnished.

Dr. SPITTAL first gives a description of the stethoscope, and the mode in which it ought to be applied; he then points out the physical signs observed in the lungs and pleuræ by auscultation. The third chapter, on "auscultation of the heart's action in health," is divided into four sections: 1, on the impulse and sounds of the heart's action; 2, on the rhythm or order of contraction of the different parts of the heart; 3, on the cause of the first sound heard during the action of the heart; 4, on the cause of the second sound produced by the action of the heart. The next subjects considered are auscultation of the heart in disease, and of the arteries in health and in disease; auscultation of the sounds yielded by the fœtal circulation; and on the diagnosis of fractures by the same means.

Forty-four cases are related, illustrative of the signs made known by auscultation. The cases are generally given in full, without the treatment; but the remarks upon them are confined to the correspondence between the auscultation and percussion and the morbid appearances. In nearly all the cases related, the author had the opportunity of verifying the correspondence between the morbid appearances and the stethoscopic signs. The student in auscultation will find this work a very useful and convenient guide.

---

AUR. COR. CELSUS *on Medicine, in eight Books, Latin and English. Translated from* L. TARGA'S *Edition; the Words of the Text being arranged in the Order of Construction. To which are prefixed, a Life of the Author, Table of Weights and Measures, with explanatory Notes, &c. Designed to facilitate the Progress of Medical Students.* By ALEXANDER LEE, A.M., Surgeon. In two vols. Vol. I.—8vo. pp. 318. Cox, London.

Too many facilities cannot be afforded to medical students for the perfect comprehension of the elegant writings of CELSUS, as they are now selected as one of the tests by which their classical attainments are determined, when they present themselves for examination. We know some instances in which candidates have been rejected for their entire deficiency in classic lore, who were fully equal to have passed through a severe scrutiny on professional subjects, with satisfaction to their examiners and credit to themselves: and some even who could read Horace or Virgil with perfect ease, have failed entirely when they were called upon to translate a page or two of Celsus. But little labour is required to overcome this difficulty, however slender may have been their stock of school instruction. Latterly, several editions of Celsus have been published, suited for readers possessing different degrees of classical attainments; but for the student whose knowledge of the Latin language is very limited, we strongly recommend this edition by Mr. Lee. An Ordo Verborum is given, which will very materially lighten the labour of study. The translation is as close to the original as the idioms of the two languages will permit, and the necessary elliptical words are generally rendered in *italics;* notes explanatory of the pharmaceutical preparations, &c. mentioned in the original, will be given at the end of the second volume. This is a very important and useful addition.

It may by some, perhaps, be thought trifling to mention the embellishments of such a work; but we are convinced that an elegant type and good paper are not merely attractive to the eye: many a student would work with pleasure at a book that is "got up" in the style of this edition, who would quickly yawn over pages thickly printed in small type and upon bad paper.

---

*Operative Surgery.* MAINGAULT'S *Illustrations of the different Amputations performed on the Human Body, represented by Plates designed after Nature; with Alterations and Practical Observations.* By WILLIAM SANDS COX, Lecturer on Anatomy and Surgery at the Birmingham School of Medicine, and Surgeon to the General Dispensary.—Folio. Longman, London.

THE objects of this work are to reduce to a small number the general rules to be observed in amputations; to represent the different modes of operating, by lithographic engravings designed after nature, the form and extent of the wounds, the principal points to be observed, and to enumerate every circumstance appertaining to the subject with the greatest simplicity. In the case of a difficult amputation, M. MAINGAULT has placed the member amputated in juxtaposition with the same member in its natural state, so that the different processes of the bone, and the depressions which are to be avoided by the knife, may be very readily distinguished; and to add to these advantages, on both are represented, by lines marked out in various directions, the precise points at which the operations may be performed. These lines not only determine the choice of situation, but, being traced over the contiguity and continuity of the bones, indicate the extent and the direction of the incisions, together with the connexion and relative position of the different parts; thus removing the difficulties with which these operations are sometimes surrounded. The different modes of operating are represented by well-executed lithographic engravings. This work will be found very useful to students and young surgeons.

In justice to Mr. Cox, we must remark that he has assumed a higher office than that of a mere translator: he "has been in the habit of demonstrating the proposed operations to his surgical class, and, being convinced of the fidelity of the descriptions," he offers the work to the student, with alterations and considerable additions.

Maingault's French edition was presented to the Institute of France, and reported on by PELLETAN and PERCY: their opinion of the plates was thus expressed, "*Tout y est d'une grandeur naturelle, la pureté et la correction en sont remarquables.*"

